# Forensic Implications of the Discrepancies Caused between NGS and CE Results by New Microvariant Allele at Penta E Microsatellite

**DOI:** 10.3390/genes14051109

**Published:** 2023-05-18

**Authors:** Balázs Kocsis, Norbert Mátrai, Balázs Egyed

**Affiliations:** 1 Department of Genetics, Hungarian Institute for Forensic Sciences, 1087 Budapest, Hungary; 2 Department of Genetics, ELTE Eötvös Loránd University, 1117 Budapest, Hungary

**Keywords:** Precision ID GlobalFiler NGS STR Panel v2, next generation sequencing, Penta E microsatellite, microallele variant, genotype discrepancy, forensics

## Abstract

Examination of STR markers using the MPS technology is becoming more common in forensic genetics, but scientists still have insufficient experience in dealing with ambiguous results. However, it is always essential to resolve discordant data if we want to use the technology as an accredited method in routine forensic casework. During the internal laboratory validation of the Precision ID GlobalFiler NGS STR Panel v2 kit, we observed two discrepant genotypes at Penta E locus compared to the previous capillary electrophoresis results. Each NGS software that we applied (i.e., Converge, STRaitRazor and IGV) returned the same 12,14 and 12,16 genotypes in the two samples, respectively, instead of the 11.3,14 and 11.3,16 genotypes previously observed with CE (*Capillary electrophoresis*) typing. In the case of the length variant 11.3 alleles, traditional Sanger sequencing confirmed a complete twelve repeat unit structure in both samples. However, after sequencing was extended to the flanking regions of the variant alleles, sequence data revealed a two-bases GG deletion downstream of the last TCTTT repeat motif in the forward strand. The determined allele variant has not been previously reported in the scientific literature and highlights the need for a careful evaluation and thorough concordance studies before using NGS STR data in forensic cases.

## 1. Introduction

The capillary electrophoresis based STR analysis (CE) was and still is the standard method used for personal identification in forensic genetics [1]. However, the use of massively parallel sequencing (MPS) will provide a major boost to the field as the MPS STR analysis has many advantages over the traditional CE methods; for instance, it can define the complete nucleotide sequence of an allele genotyped encompassing the repeat units and the flanking regions as well [2]. Although the discrimination power of the CE-based alleles is continuously growing with the introduction of new expanded multiplexes, their complexities caused by the ever-growing amplicon sizes and the used dyes may anticipate the limits of the traditional technology. Hence, there is no surprise that the willingness of the scientific community to use the new MPS technologies is increasing. Today, it is no question that by applying MPS STR typing in forensics, the highest discrimination power can be achieved in human identification. In addition, this modern technology is not limited to the repeat polymorphism, because single nucleotide polymorphisms (SNPs) can also be targeted together with the repeat numbers. Several studies demonstrated that MPS STR typing, compared to CE-based analysis, obtained an increase in allele variability [3]. In 2016, because of the numerous newly discovered allele variants, considerations on minimal nomenclature requirements for forensic MPS STRs and the minimal requirement of data storage that laboratories should follow when adopting MPS of STRs were published by the DNA commission of the ISFG [4]. In addition to the recommendations, the online STRidER database (STRs for Identity ENFSI Reference Database) has been created [5], which was an enlarged and improved version of the preceding ENFSI STRBase. Two years later, Phillips et al. released an expanded, enhanced and dynamically revised forensic STR Sequence Guide [6]. In these early papers, principally, the sequence-specific allele variants detected in the repeat regions were described and published. However, variant sequences in the flanking regions cannot be neglected at all, which could be up to 20% of the newly described allele variants of the total SNP pool in Illumina chemistry and about 5% in Ion Torrent chemistry [7,8,9,10,11,12].

However, besides the advantages of this new method, it is necessary to discuss the challenges regarding sequence-based analyses, such as the backward compatibility of NGS data to CE results during a concordance validation. Some studies in which the Precision ID GlobalFiler NGS STR Panel v2 was used reported two types of discordances between CE and GlobalFiler panel data. Discordant genotypes were mostly due to observed base pair deletions in the flanking regions. In three cases, an allele 2.2 dropout was observed at Penta D locus during sequence evaluation of MPS STR using the Converge software (Thermo Fisher Scientific) where preceding CE genotypes were 2.2,11; 2.2,9; and 2.2,14, respectively [7]. Further analyses of raw data and FASTQ files using STRaitRazor v3 [13] proved the presence of allele 2.2 with a 13 bp deletion in the 5′ flanking region (rs119098807) in each case. Another study detected a 2 bp deletion at the D19S433 locus located in the 5′ flanking region (rs745607779) that was revealed via the IGV software (Integrative Genomic Viewer) [14] and this resulted in a 13.2 allele in MPS STR typing compared to allele 14 determined using CE [10]. Other cases demonstrated very low read count of some alleles during sequence evaluation and due to the extremely unbalanced amplification during library construction, allelic dropouts or 1–3 bp insertions had been observed which belonged to X1-3 type error, according to a previous study [12]. In one case, Converge software did not detect a 1 bp insertion before the first complete repeat unit at the D2S441 locus but the analysis of the raw data and the FASTQ file showed that the result was in accordance with CE data and revealed the questioned 13.1 allele [7].

During our internal laboratory validation, the Precision ID GlobalFiler NGS STR Panel v2 each genotype determined using the new MPS STR method was in accordance with the previous CE results, excepting two cases, in which allelic discrepancies were observed at Penta E locus between NGS and CE-based data. Instead of the prior 11.3,14 and 11.3,16 genotypes revealed via CE typing, NGS resulted in 12,14 and 12,16 genotypes, respectively.

## 2. Materials and Methods

For concordance testing during internal validation, our laboratory elimination database was used for 60 adult DNA samples with known family relations and with approved written consent. DNA was extracted from buccal swabs using EZ1 Advanced XL instrument with EZ1&2 DNA Investigator Kit according to the manufacturer’s recommendations (Qiagen, Hilden, Germany). Extracted genomic DNA was quantified using the Quantifiler Trio DNA Quantification kit (Thermo Fisher Scientific, Waltham, MA, USA) in the 7500 Real-Time PCR System (Applied Biosystems, Waltham, MA, USA) and was analysed using the HID Real-Time PCR Analysis Software 1.2 (Thermo Fisher Scientific). For conventional STR typing, samples were amplified using the PowerPlex Fusion 6C and the PowerPlex 18D Systems according to the manufacturer’s recommendations (Promega, Madison, WI, USA). The GeneAmp PCR System 9700 thermal cycler was used for amplification and the electrophoresis was run on an ABI 3500xl Genetic Analyzer (Thermo Fisher Scientific). The electropherograms were analysed using GeneMapper ID-X software v1.6 (Thermo Fisher Scientific).

For massive parallel sequencing, the library preparation was performed in 500 pg DNA template using the GlobalFiler NGS STR Panel v2 and the Precision ID Chef DL8 kits (Applied Biosystems, Waltham, MA, USA) with total reaction volumes of 15 µL. Each libraries contained a total mix of eight samples which were quantified using the Library TaqMan Quantification kit (Thermo Fisher Scientific) in the 7500 Real-Time PCR System. The E. coli DH10B control library was used as a quantification standard (Thermo Fisher Scientific). The libraries were diluted to 50 pM and then four libraries were mixed in equal proportions (6.25 µL per library) to a final volume of 25 µL for one sequencing run on Ion 530 chip. The Ion S5 Precision ID Chef Reagents were used for the automatic template preparation according to the manufacturer’s recommendations (Thermo Fisher Scientific). MPS was performed on the Ion S5 System using the Ion S5 Precision ID Sequencing Kit following the manufacturer’s instruction (Thermo Fisher Scientific). Sequence data were generated and evaluated using the Torrent Suite Software v5.10 (Thermo Fisher Scientific). For reference alignment, the Homo sapiens hg19 genome was applied (GCF_000001405.40). The HID STR Genotyper plugin v2.2 (Thermo Fisher Scientific) was used for STR typing at default thresholds excepting the analytical threshold (AT) and the stochastic threshold (ST) at some loci where the applied threshold values had been generated in course of the internal validation process.

For PCR and Sanger sequencing of Penta E alleles, unlabelled oligonucleotide primer sequences were downloaded from STRBase (https://strbase.nist.gov, accessed on 7 February 2023). PCR amplifications were performed on a GeneAmp 9700 PCR instrument with 0.5 ng template DNA using AmpliTaq Gold DNA Polymerase chemistry (Applied Biosystems) in 25 µL of total PCR volume. PCR conditions were denaturation for 10 min at 96 °C, then 94 °C at 20 s, 56 °C at 10 s, and 72 °C at 30 s for 32 cycles, and final extension at 72 °C for 10 min. PCR products were run on 3% agarose gel (SeaKem Agarose, Lonza, Basel, CH) to separate and visualise the heterozygous fragments. The two separate bands in each PCR product were excised and purified using Zymo Research Clean and Concentrator-25 Kit (Zymo Research, Seattle, WA, USA). In order to better sequence performance, the purified DNA fragments were reamplified and repurified as previously described. DNA sequencing were performed using BigDye Terminator v1.1 Cycle Sequencing Kit (Applied Biosystems) in a 10 µL total volume with both forward and reverse primers. After cleaning up (Zymo Research DNA Sequencing Clean-up Kit), the reactions were analysed on an ABI Prism 3130xl Genetic Analyzer instrument and sequence data were evaluated using SeqScape v3.0 (Thermo Fisher Scientific) and Sequencing Analysis Software v6.0 (Thermo Fisher Scientific) and aligned to the Homo sapiens hg19 reference genome.

## 3. Results

The CE-based PowerPlex Fusion 6C multiplex-PCR kit has already been validated earlier in the laboratory for routine casework. During the validation process, two samples in the elimination database carried a microvariant allele 11.3 at Penta E locus. The observed heterozygous genotypes with the microvariant allele were 11.3,16 and 11.3,14, respectively, and results were also confirmed using the PowerPlex 18D multiplex system. Although both CE-based multiplex-PCR kits were produced by the same manufacturer, the detected fragment sizes of the same alleles differed considerably between the two systems (Figure S1A,B). The approximately ten bases difference in size of the identical alleles can be due to the various primers applied, or due to adopted linker sequences. Unfortunately, there was no further information from the manufacturer regarding the oligos. The genetic relationship between the two samples harbouring the variant alleles were discovered and the mother/daughter descent was proven later via further DNA analyses.

During the recent internal validation of the Precision ID GlobalFiler NGS STR Panel v2 kit on Ion S5 System platform, we received, however, the non-variant 12,16 and 12,14 genotypes at Penta E in the two samples, respectively, instead of the previously observed CE-based alleles. The Converge Software 2.2 was used primarily to evaluate NGS data in the laboratory. NGS genotypes in both samples were determined by normal read count for each allele (Figure S2A). All the other loci and genotypes in the two samples generated via the Converge were concordant with previous CE results. For secondary NGS analysis, FASTQ files were analysed with the freely available STRaitRazor v3 software which confirmed the same non-variant allele 12 in the samples (Figure S2B). Furthermore, the Binary Sequence Alignment/Map (BAM) files from these samples were analysed using the IGV v2.12.2 software, which determined the same non-variant allele 12 in both genotypes instead of the CE detected 11.3 microvariant (Figure S2C). IGV reads clearly revealed twelve repeat units in the samples, and neither SNP nor indel sites were detected in the approximately 160 bp long sequence read that covered the complete repeat structure and partly the flanking regions of the Penta E locus.

**Figure 1:**
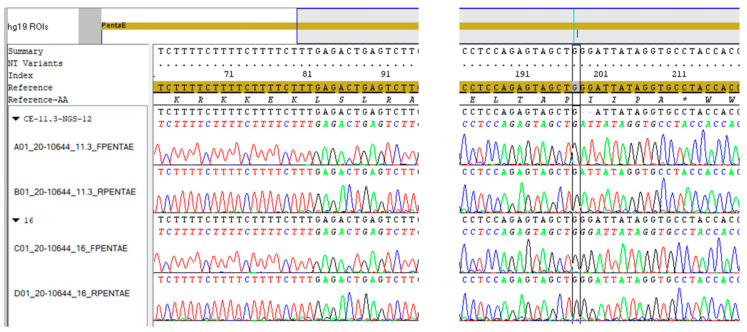
Sanger sequencing of alleles 12 and 16 at Penta E locus including alignment to human genome assembly hg19. Both strands (A01 and B01 samples) revealed 12 repeat motifs (in here shown the last four TCTTT) of the discordant allele 11.3, however, in the flanking region of the discordant allele 12 (upper two lanes), there were two-base-pair GG deletion, 116–117 bases, upstream of the last TCTTT repeat motif in both strands (after the black box at the 198–199 index positions). At allele 16 (C01, D01), there was no discrepancy.

To resolve the discrepant genotypes observed between the two methods, we decided to make further sequence analyses on the samples. For this purpose, traditional Sanger sequencing was the good choice to determine the accurate sequence of the PCR-amplified alleles. After separating the questioned alleles using electrophoresis, DNA fragments were isolated from the gel, purified and reamplified, and Sanger sequencing were conducted on each isolated samples, both on forward and reverse strands. The evaluated DNA sequences were aligned to the Penta E locus of the human hg19 reference genome (Penta E: chr15-hg19-97.374.245-97.374.269). The sequences of the variant alleles were in accordance with the complete repeat structure motifs, i.e., there were no base pair insertion/deletion variance observed in the repeat sequences at all. The sequenced repeat numbers of each allele were in accordance with the genotypes determined using MPS STR and CE. In case of the variant 11.3 alleles, the sequence data confirmed the complete twelve repeat unit structure in both samples, that corresponded with previous NGS sequence results. In parallel, the 14 and 16 repeat motifs have been identified in the homologous alleles. However, for PCR and Sanger sequencing, relatively long size DNA fragments were amplified (414 bp long fragment in case of allele 12), that covered the whole domain of the locus and made it possible to analyse the repeat structure of the nearby flanking regions in a more wide range. The extended sequencing of the flanking regions in discordant 11.3 allele revealed a two-bases GG deletion, 116–117 bases, downstream of the last TCTTT repeat motif in the forward strand. This dinucleotide deletion has been observed in both samples harbouring the microvariant 11.3 alleles, and the detected variant site was located at 203–204 bp distance from the 3′ end of the forward primer at the 97.374.386–97.374.387 chromosome positions on chromosome 15 aligned to the reference human genome hg19 (Figure 1).

Several searches have been conducted in the various important electronic available sequence databases to check the existence of this variant allele sequence (i.e., GenBank, STRBase, STRSeq BioProject, 1000 Genomes Browser, and UCSC Genome Browser). Some sequences in the databases revealed substitutions on this site, and there were deletions, insertions, or substitutions observed at the closest bases, but there was no similar dinucleotide deletion that we determined. Additionally, no references for this allele variant in the scientific literature were found.

According to the manufacturer’s application guide for the Precision ID GlobalFiler NGS STR Panel v2 kit, the amplicon read sizes at Penta E locus were settled from 168 to 273 bases long-range depending on the sequenced allele. The amplicon read lengths also included the two primer oligos that were approximately 30 nt long. However, during the NGS data evaluation we were unable to determine fragments longer than 160 bp long in the raw data using IGV. This means that a considerable part of the allelic sequence reads fell out of the detected range. Indeed, inspecting the aligned IGV sequences, maximum 60 bp long fragments were readable at the forward strand downstream region of the last TCTTT repeat, and only 35 bp stretch upstream were readable from the starting repeat in the flanking region. Based on the Sanger sequencing results and considering the NGS amplicon sizes, the identified new mutation, i.e., GG deletion, must located in the downstream flanking region of the forward strand far away enough from the repeat structure that might have caused the kit amplicons did not cover the mutated site during library preparation. This phenomenon led to ignore the mutated Penta E alleles during NGS data evaluation and resulted in the misgenotyping of the two samples using the Precision ID GlobalFiler NGS STR Panel v2 kit that ultimately caused the discrepancy between NGS and CE-based results.

Based on the literature, similar microvariant sites have been observed at almost all forensically relevant autosomal STRs, when usually base deletion in the flanking region far away from repeat structure was the reason for a discordant result. Nevertheless, according to the STRidER database (https://strider.online/, accessed on 7 February 2023), only three variant sites were found until now in the Penta E sequence downstream of the last TCTTT repeat unit. The reviewed rs188309642 and rs537369792 SNP positions were identified in African population samples, and the rs759423627 SNP in a European sample pool. However, the indel variation described in our study at 116–117 nucleotides downstream from the last repeat unit, just falls outside of the database sequence deposited in STRidER which ends at 115 nt position (GRCh38 coordinate: 96831154). The sequence of the new observed Penta E 11.3 allele variant has been annotated and deposited in the GenBank (https://www.ncbi.nlm.nih.gov/genbank/, accessed on 7 February 2023) with accession number OP700292.

## 4. Discussion

In this study, we described an STR discrepancy case between NGS and traditional CE data that could not have been resolved using only different NGS software packages, and neither by re-analysis of the samples. Usually, microvariant alleles falling between two repeat units in size (such as 11.3 observed at Penta E locus) can be obtained in different ways using CE: as an insertion or deletion in the tandem repeat region, or as an indel variant in the flanking sequences that does not affect the repeat structure but still investigated using the muliplex-PCR systems. To determine the reason behind the genotype discrepancy in this case, an extended sequence analysis has been performed on the samples in question. Sanger sequencing of the variant alleles proved that they carry a GG deletion in the flanking region far away from the repeat stretch that resulted in the observed 11.3 intermediate alleles which were undetected via the applied MPS analysis software.

Nevertheless, in forensic genetics the fragment length and repeat unit based allelic nomenclature is still accepted, but there is an increasing demand on comprehensive STR nomenclature systems to adopt MPS STR data in order to ensure compatibility with MPS and with traditional CE data [4]. The intermediate alleles observed in this study were confirmed as having complete twelve repeat unit structure, therefore calling them as 12-2 or 12var corresponds to current nomenclature. It has to be also noted that relevant genetic variation outside the common repeat regions of STR sequences should be stored in databases as sequence strings that include flanking sequences according to the most recent recommendation [4].

There were several reasons why we performed this deep analysis of the variant allele: (i) If an evidence sample carrying allele 11.3 at Penta E was genotyped using capillary electrophoresis but reference samples were genotyped using only NGS, an exclusion would be considered on this locus in a casework; (ii) The sequenced and annotated allele variant has been published and deposited in open databases that makes this observation available to the scientific community; and (iii) The results provide a clear explanation of the observed allele discrepancy to any another lab that would detect similar phenomenon. If a forensic lab using NGS for STR typing is aware of the potential pitfalls of the method used, similar issues can be avoided during interpretation of expert reports. However, the correct repeat numbers that were revealed using the NGS technology prove the usability of the technique in routine casework. In addition, our study highlights the necessity to consider applying several software packages or even traditional molecular methods for the evaluation of questionable NGS results or discordant genotypes.

## Data Availability

The data presented in this study are available on request from the corresponding author. The data are based on internal laboratory elimination database.

## Supplementary Materials

The following supporting information can be downloaded at: https://www.mdpi.com/article/10.3390/genes14051109/s1, Figure S1: Discordant variant allele (11.3) at Penta E locus in case of PowerPlex Fusion 6C System (S1A) and PowerPlex 18D System (S1B); Figure S2: NGS results of the microvariant 11.3, 16 genotype at Penta E with different softwares.
