# Supplementary material for: Forensic Implications of the Discrepancies Caused between NGS and CE Results by New Microvariant Allele at Penta E Microsatellite
Source: Genes (Basel). 2023 May 18;14(5):1109. doi: 10.3390/genes14051109 (PMC10218238; doi:10.3390/genes14051109)
Supplement: Supplementary file 1 [file genes-14-01109-s001.zip › genes-2380938-supplementary.docx]

A)


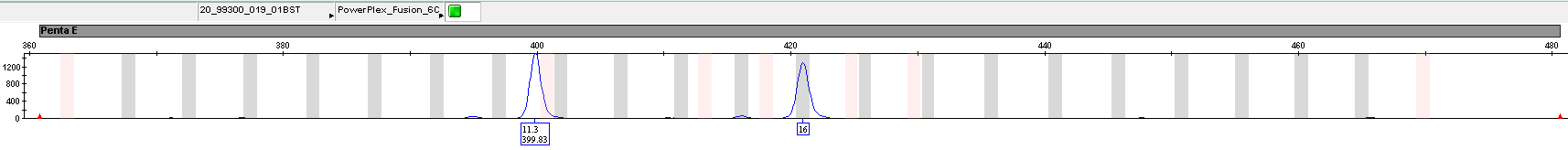


B)


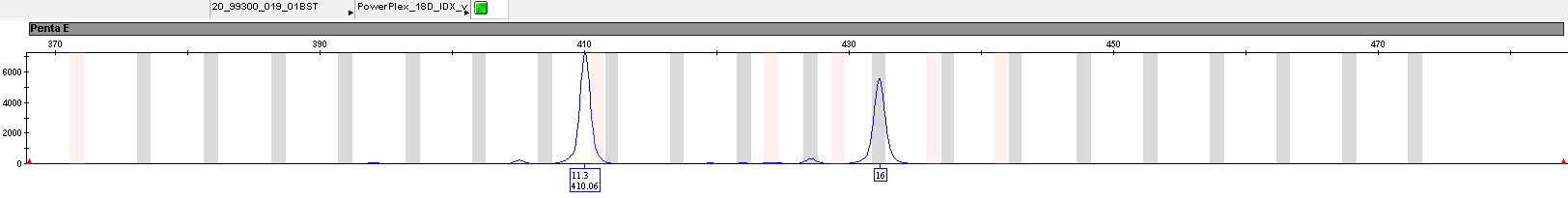


**Figure S1.** Discordant variant allele (11.3) at Penta E locus in case of PowerPlex Fusion 6C System (**A**) and PowerPlex 18D System (**B**)

A)


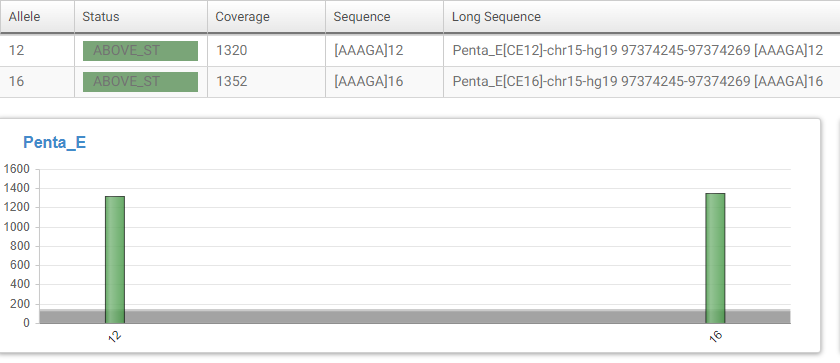


B)


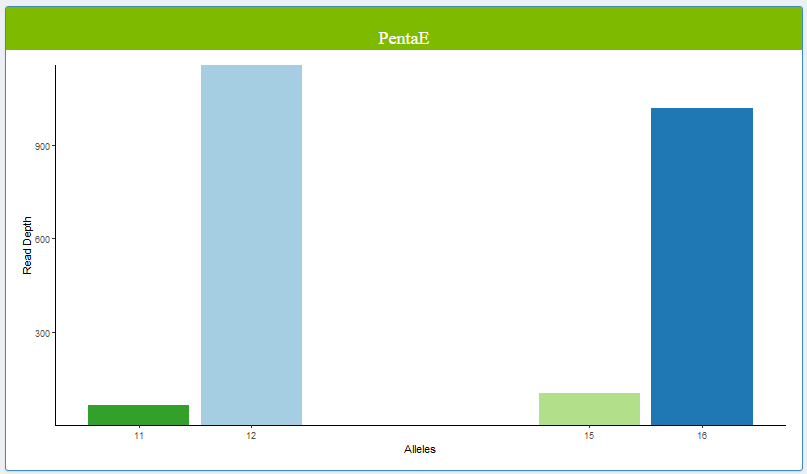


C)


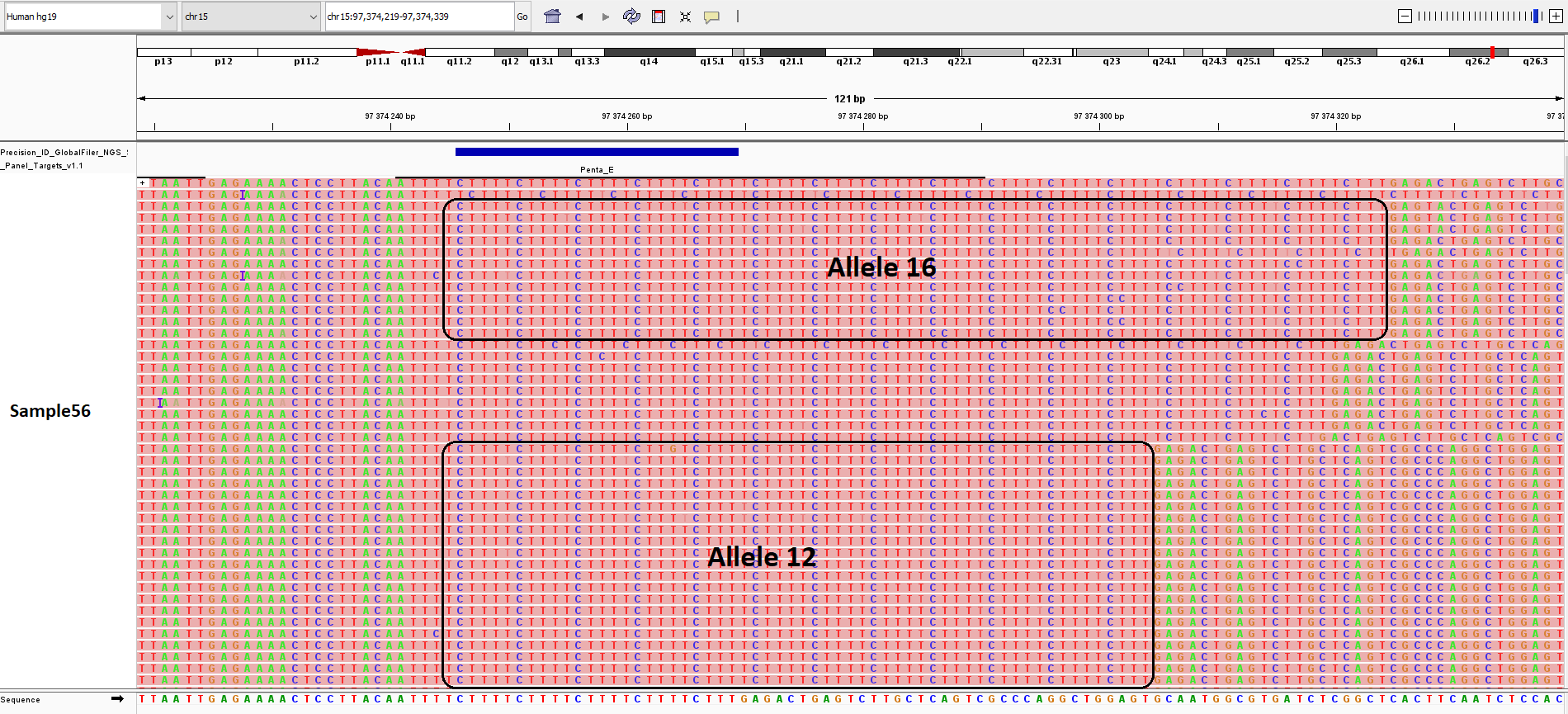


**Figure S2.** NGS results of the microvariant 11.3,16 genotype at Penta E with different softwares. (**A**) Converge software v2.2 result including short and long sequence data with coverage. (Because of factory settings only reverse sequence is visible.) (**B**) STR genotyping with STRaitRazor v3 software including bar chart representation of allele calls and coverage. (**C**) Analysis of BAM files using IGV 2.12.2 software including graphical alignment relative to human genome assembly GRCh37 (hg19). IGV reads clearly detect 12 and 16 repeat motifs in the samples (black boxes) without any SNPs or InDels in the repeat structure or in the flanking regions.
